# The Retinoic Acid Receptor-α mediates human T-cell activation and Th2 cytokine and chemokine production

**DOI:** 10.1186/1471-2172-9-16

**Published:** 2008-04-16

**Authors:** Harry D Dawson, Gary Collins, Robert Pyle, Michael Key, Dennis D Taub

**Affiliations:** 1 Diet Genomics and Immunology Lab, United States Department of Agriculture, Beltsville, MD 20705, USA; 2 Laboratory of Immunology, Gerontology Research Center, National Institute on Aging, NIH, Baltimore, MD 21224, USA

## Abstract

**Background:**

We have recently demonstrated that all-*trans*-retinoic acid (ATRA) and 9-cis-retinoic acid (9-*cis *RA) promote IL-4, IL-5 and IL-13 synthesis, while decreasing IFN-γ and TNF-α expression by activated human T cells and reduces the synthesis of IL-12p70 from accessory cells. Here, we have demonstrated that the observed effects using ATRA and 9-*cis *RA are shared with the clinically useful RAR ligand, 13-*cis *retinoic acid (13-*cis *RA), and the retinoic acid receptor-α (RAR-α)-selective agonist, AM580 but not with the RAR-β/γ ligand, 4-hydroxyphenylretinamide (4-HPR).

**Results:**

The increase in type 2 cytokine production by these retinoids correlated with the expression of the T cell activation markers, CD69 and CD38. The RAR-α-selective agonist, AM580 recapitulated all of the T cell activation and type 2 cytokine-inducing effects of ATRA and 9-*cis*-RA, while the RAR-α-selective antagonist, RO 41–5253, inhibited these effects.

**Conclusion:**

These results strongly support a role for RAR-α engagement in the regulation of genes and proteins involved with human T cell activation and type 2 cytokine production.

## Background

Retinoids are natural or synthetic vitamin A (VA) derivatives. Retinoic acid (RA) is present in human plasma at a concentration of around 10–20 nM mainly in two stereoisomeric forms, all-*trans*-RA (ATRA, ~75%) and 13-*cis*-RA (~25%) [1]. The concentration of ATRA in most rat and human tissues is higher than the plasma concentration [2,3]. The plasma and tissue concentrations of 9-*cis *RA are at or below the limits of detection of modern methods of analysis [2,3] and very little bioconversion/isomerization to 9-*cis *RA occurs after pharmacological administration of ATRA or 13-*cis *RA. Recently, two other retinoic acid receptor interacting retinoids, all-trans-13,14-dihydroretinoic acid and beta -apo-14'-carotenal, have been identified in tissue [4,5]

Retinoids regulate the expression of more than 300 different genes through various mechanisms [6]. Predominant among the mechanisms is the ability of retinoids such as ATRA (Tretinoin^®^) and 9-*cis*-RA (Panretin^®^) to bind to at least two classes of nuclear receptors, RA receptors (RARs) and/or retinoid × receptors (RXRs) [7]. ATRA binds exclusively to RARs while 9-*cis*-RA binds to both RARs and RXRs. The affinity of 9-*cis*-RA for RXRs is about 5–10 fold higher and its transactivating potential is approximately 10–30 times higher than for RARs [8]. Moreover, ATRA is likely to exhibit some RXR-stimulating activity *in vitro *due to its conversion to 9-*cis*-RA [9]. The low plasma and tissue levels of 9-*cis*-RA have lead to a search for other possible endogenous ligands for RXRs. To date, phytanic acid [10] docosahexaenoic acid [11] and other unsaturated fatty acids [12] have been proposed as additional/alternative ligands for RXRs; whereas, beta -apo-14'-carotenal may be a naturally-generated RXR antagonist [5]

When bound with ligand, RAR/RXR heterodimers or RXR homodimers transactivate specific *cis*-elements of target genes (known as retinoic acid response elements (RAREs) or retinoid × response elements (RXREs). Each RAR or RXR has three major isoforms (α, β, or γ) which are tissue selective and developmentally regulated [7]. Individual isoforms of RARs and RXRs may interact with distinct RAREs or RXREs to regulate gene expression. Alternately RARs and RXRs may differentially interact with other transcription factors such as the nuclear receptor for 1,25 (OH)_2 _vitamin D_3 _(Vit D_3_), AP-1, CEBPB/NF-IL6 and the silencing mediator of retinoid and thyroid hormone (SMRT) to stimulate or repress gene transcription [13]. These data suggest that individual RAR and RXR isoforms may have unique functions.

The role of specific retinoid receptors in the differential regulation of immune responses is an area of active interest. This is particularly true for T cell-mediated immunity. The role of RAR-γ in the induction and RAR-α/RXR-γ in the inhibition of thymocyte apoptosis has been extensively characterized [14]. However, the role of specific retinoic acid receptors in peripheral T cell function is largely unknown. Several groups have demonstrated that expression of RAR-α RNA and protein increases after T cell activation [15,16]. In contrast, RAR-γ and RXR-α expression decrease during T cell activation [17,18]. While the precise biological role of these differentially expressed retinoic acid receptor isoforms by activated human peripheral T cells is currently unknown, several research groups have explored the role of different retinoid receptors in the regulation of T cell-derived cytokines such as interferon-γ (IFN-γ) and interleukin-4 (IL-4). IFN-γ and IL-4 are produced in a reciprocal fashion by T cells and are associated with T helper type 1 (Th1) and Th2-associated responses, respectively.

Reports on the effects of specific retinoic acid receptors on IFN-γ production by T cells appear contradictory. In one study, ATRA significantly downregulated IFN-γ promoter activity in RAR-α-transfected Jurkat cells [19] via interaction with a negative response element putatively identified as USF1. Other reports described the ability of the RAR-α-selective retinoid, Am80 [20] to inhibit IFN-γ from antigen-stimulated mouse T cells [21,22]. However, a another report demonstrated no effect of Am80 on IFN-γ production by murine Th1 clones [23], while the pan-RAR/RAR-β-selective retinoid, CH55 [24], was more potent than either ATRA or 9-*cis *RA in inhibiting IFN-γ production from murine Th1 clones [25]. Finally, *in vitro*, antigen-specific, IFN-g synthesis was inhibited by 9-*cis*-RA and a RXR-selective retinoid but not by ATRA or a RAR-selective retinoid [26]. In murine memory T cells, 9-*cis *and a RXR-selective retinoid inhibited IL-4, IL-5 and IFN-γ production [27] Finally, a single study showed that 9-*cis*-RA reduced IL-13 mRNA from *ex-vivo *antigen-stimulated murine T cells [28].

A number of *in vivo *studies conducted to determine the role (s) of specific retinoic acid receptors on Th1- or Th2-mediated pathologies have also yielded conflicting results. Systemic administration of RAR-α-selective retinoids have been shown to inhibit Th1-associated immune responses such as delayed type hypersensitivity (DTH) [21,29], the progression of experimental arthritis [23] and skin allograft rejection [30]. In contrast, systematic administration of an RXR antagonist decreased production of IL-4, IL-10, and IL-13, and increased IFN-γ production and increased inflammation in a mouse model of allergic lung inflammation [31]. Additionally, mice expressing a hypomorphic mutation in the RXR-α gene have an exaggerated Th1 immune response but a relatively normal TH2 response [32]. Finally, mice engineered to express reduced amounts of RXR-α in T cells showed no difference in polyclonally-stimulated IL-5, IL-5 and IFN-g production, *ex vivo *naïve to memory Th1 or TH2 development and only a small increase in IL5 and a small decrease in IFN-γ production from memory cell [27].

Despite their potential clinical utility, a very limited number of studies have examined the comparative ability of retinoids such as ATRA, 9-*cis *RA, 13-*cis *RA, and 4-HPR to modulate Th1 cell or Th2 cell differentiation. One *in vitro *study indicated that 13-*cis*, ATRA, and 9-*cis*-RA were equipotent in inhibiting IFN-γ production from murine Th1 cells [25]. Similarly, our own previous work suggested that ATRA and 9-*cis *RA were equipotent in modulating Th2-associated responses in human T cells [33]. An additional report demonstrated that 13-*cis *RA and 4-hydroxyphenylretinamide (4-HPR), a synthetic retinoid, equally inhibited the progression of experimental autoimmune encephalomyelitis (EAE) in mice [34]. Previous studies have demonstrated that the balance of Th1 to Th2 T cells plays a role in the progression of this disease. Although this limited data would suggest that ATRA, 9-*cis *RA 13-*cis *RA and 4-HPR may be equal in their Th2-inducing activity, their potential differences in receptor selectivity and the differential ability of receptor-selective retinoids to modulate Th2-development in murine models, indicate that these retinoids may differ in their ability to influence human Th1/Th2 development. In the current report, we have examined the comparative ability of ATRA, 9-*cis *RA 13-*cis *RA and 4-HPR as well as RAR-α-selective agonists and antagonists to modulate T cell activation as well as promotion of a Th2-associated cytokine response. Our data supports a role for RAR-α engagement in T cell activation and the preferential expression of Th2-associated cytokines

## Results

### Differential expression of type 1- and type-2-associated cytokines and cell surface activation markers by various retinoid compounds

**Figure 1:**
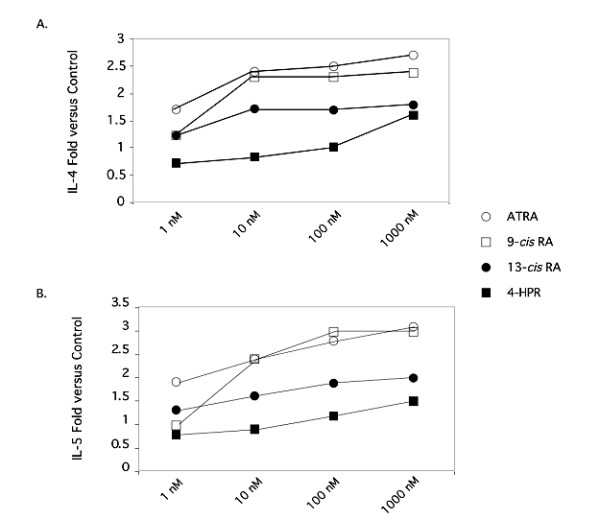
**The effects of various clinically-utilized retinoid compounds on Th2-associated cytokine production by anti-CD3e-activated PBMCs**. Supernatants of anti-CD3e-activated PBMCs treated with EtOH or 10^-9 ^to 10^-6 ^M ATRA (○), 9-*cis*-RA (□), 13-*cis*-RA (●), or 4-HPR (■) for 48 h were examined for IL-4 or IL-5. The values shown represent the average fold change obtained from 4 donors.

**Figure 2:**
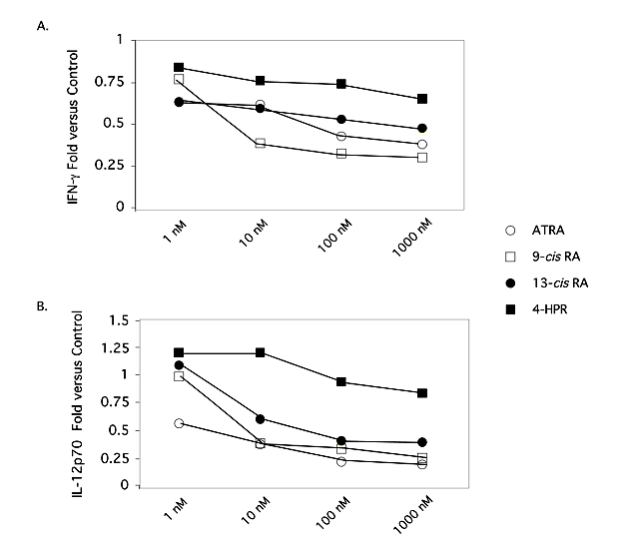
**The effects of various clinically-utilized retinoid compounds on Th1-associated cytokine production by anti-CD3e-activated PBMCs**. Supernatants of anti-CD3e-activated PBMCs treated with EtOH or 10^-9 ^to 10^-6 ^M ATRA (○), 9-*cis*-RA (□), 13-*cis*-RA (●), or 4-HPR (■) for 48 h were examined for IFN-γ or IL-12 protein levels by ELISA analysis. The values shown represent the average fold change obtained from 4 donors.

Initial studies focused on comparison between the retinoid compounds, ATRA and 9-*cis*-RA, and the clinically utilized retinoids, 13-*cis*-RA and 4-HPR. The results shown in Table 1 and Figure 1 demonstrate the ability of the retinoids, ATRA, 9-*cis*-RA and 13-*cis*-RA, to significantly stimulate production of IL-4 from anti-CD3 mAb-stimulated PBMC in a dose-dependent fashion. While ATRA and 9-*cis*-RA were equipotent in this regard, 13-*cis*-RA was about 10 fold less active. 4-HPR was slightly inhibitory at low doses and stimulatory only at the highest dose (1 μM). Similarly, the effects of retinoids on IL-5 (Table 1 and Figure 1), and IL-13 (data not shown) production were remarkably similar to that of IL-4. They had no effect on IL-10 (data not shown). For Th1-associated cytokines, 9-*cis*-RA was approximately 10 times more effective than ATRA at inhibiting IFN-γ production (Table 2 and Figure 2). 13-*cis*-RA and ATRA were equipotent and 4-HPR was approximately 1,000 fold less effective. For IL-12p70, ATRA was more effective than 9-*cis *and 13-*cis *RA at 1 nM but was equipotent at 10–1000 nM.

**Table 1:** ATRA, 9-cis RA and 13-cis RA significantly increase the production of Th2-associated cytokines from anti-CD3-stimulated PBMC cultures in a dose-dependent fashion.

**IL-4**	**Fold vs Control ± SE (n = 3)**			
	**Doses (nM)**			

	**1**	**10**	**100**	**1000**

**Retinoid**				
**ATRA**	1.7 ± 0.55	2.4 ± 0.40	2.5 ± 0.48	2.7 ± 0.64
**9-cis-RA**	1.2 ± 0.05	2.3 ± 0.38	2.3 ± 0.57	2.4 ± 0.65
**13-cis-RA**	1.2 ± 0.13	1.7 ± 0.25	1.7 ± 0.42	1.8 ± 0.30
**4-HPR**	0.7 ± 0.11	0.8 ± 0.18	1.0 ± 0.13	1.6 ± 0.45
				
**2-way ANOVA**				
Dose	*p = *0.02			
Retinoid	*p *= 0.0003			
				
**Tukey Kramer**				
Dose	*p *< 0.05	1 nM vs 100 nM		
		1 nM vs 1000 nM		
Retinoid	*p *< 0.01	4-HPR vs ATRA		
	*p *< 0.01	4-HPR vs 9-*cis*-RA		

**IL-5**	**Fold vs Control ± SE (n = 3)**			

	**Doses (nM)**			

	**1**	**10**	**100**	**1000**

**Retinoid**				
**ATRA**	1.9 ± 0.41	2.4 ± 1.0	2.8 ± 1.1	3.1 ± 1.3
**9-cis-RA**	1.0 ± 0.10	2.4 ± 1.0	3.0 ± 0.85	3.0 ± 1.1
**13-cis-RA**	1.3 ± 0.31	1.6 ± 0.45	1.9 ± 0.67	2.0 ± 0.84
**4-HPR**	0.8 ± 0.05	0.9 ± 0.26	1.2 ± 0.40	1.5 ± 0.30
				
**2-way ANOVA**				
Dose	*p *= 0.07			
Retinoid	*p *= 0.005			
**Tukey Kramer**				
Retinoid	*p *< 0.05	4-HPR vs 9-*cis *RA		
	*p *< 0.01	4-HPR vs ATRA		

**Table 2:** ATRA, 9-cis RA and 13-cis RA significantly decrease the production ofTh1-associated cytokines from anti-CD3-stimulated PBMC cultures in a dose-dependent fashion.

**IFN-γ**	**Fold vs Control ± SE (n = 3)**			
	**Doses (nM)**			

	**1**	**10**	**100**	**1000**

**Retinoid**				
**ATRA**	0.63 ± 0.04	0.61 ± 0.05	0.43 ± 0.13	0.38 ± 0.10
**9-cis-RA**	0.76 ± 0.06	0.38 ± 0.09	0.32 ± 0.06	0.30 ± 0.05
**13-cis-RA**	0.64 ± 0.11	0.59 ± 0.04	0.53 ± 0.04	0.47 ± 0.09
**4-HPR**	0.83 ± 0.11	0.75 ± 0.07	0.73 ± 0.02	0.65 ± 0.03
				
**2-way ANOVA**				
Dose	*p *= 0.0001			
Retinoid	*p *< 0.0001			
				
**Tukey Kramer**				
Dose	*p *< 0.01	1 nM vs 100 nM		
		1 nM vs 1000 nM		
Retinoid	*p *< 0.01	4-HPR vs ATRA		
		4-HPR vs 9-*cis *RA		
		4-HPR vs 13-*cis *RA		

**IL-12p70**	**Fold vs Control ± SE (n = 3)**			

	**Dose (nM)**			

	**1**	**10**	**100**	**1000**

**Retinoid**				
**ATRA**	0.57 ± 0.18	0.40 ± 0.11	0.36 ± 0.13	0.27 ± 0.12
**9-cis-RA**	1.0 ± 0.12	0.39 ± 0.07	0.25 ± 0.19	0.21 ± 0.09
**13-cis-RA**	1.1 ± 0.29	0.61 ± 0.20	0.42 ± 0.14	0.41 ± 0.09
**4-HPR**	1.2 ± 0.47	1.2 ± 0.41	0.94 ± 0.26	0.84 ± 0.16
				
**2-way ANOVA**				
Dose	*p *= 0.004			
Retinoid	*p *= 0.0007			
				
**Tukey Kramer**				
Dose	*p *< 0.05	1 nM vs 100 nM		
	*p *< 0.01	1 nM vs 1000 nM		
Retinoid	*p *< 0.01	4-HPR vs ATRA		
		4-HPR vs 9-*cis*-RA		
	p < 0.01	4-HPR vs 13-*cis*-RA		

**Figure 3:**
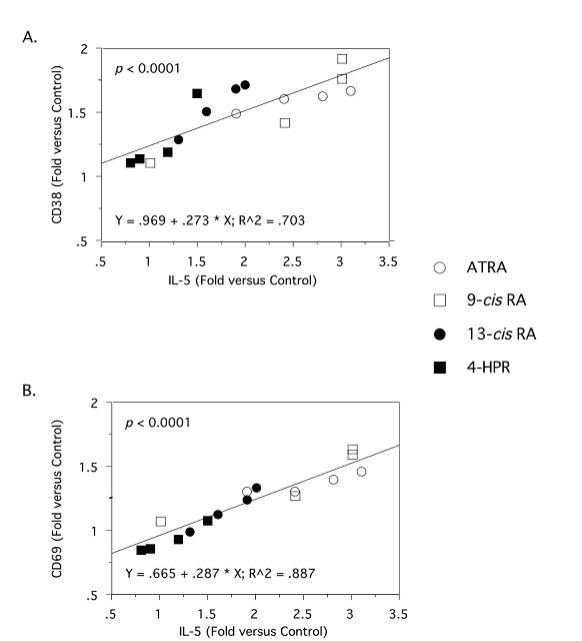
**Correlation between retinoid-induced IL-5 expression and T cell-associated CD38 and CD69 expression in anti-CD3, -activated PBMC**. IL-5 protein levels were quantitated by ELISA in supernatants of 48 h, anti-CD3,-activated PBMC treated with ETOH or ATRA (○), 9-*cis*-RA (□), 13-*cis*-RA (●), or 4-HPR (■). The values for the level of expression (mean channel number) of CD38 and CD69 on T cells were obtained by flow cytometry of cells from the same culture. Simple regression was performed on these values as described in *Materials and Methods*. Data shown is average fold change calculated from three experiments.

Given the differences in the ability of these retinoids to modulate cytokine production, we next examined whether the ability of retinoids to induce type 2 cytokine production is related to their ability to induce T cell activation. To this end, we examined the expression of the T cell activation markers, CD25, CD38, and CD69. CD69 is an early marker of T cell activation and CD38 expression is induced after T cell activation and is reportedly under the direct control of RAR-α [35]. The results in Table 3 demonstrate that ATRA, 9-*cis*-RA, and 13-*cis*-RA and to a lesser extent, 4-HPR, consistently increased the expression of CD69 and CD38. These increases were dose-dependent with following degrees of potency and efficacy observed: 9-*cis*-RA > ATRA, 13-*cis*-RA > > 4-HPR. All of the retinoids failed to increase CD25 expression above control at any concentration tested (data not shown). CD25 is part of the IL-2 receptor complex and is typically expressed as an intermediate marker of T cell activation. The relationship between type 2 cytokine expression and CD upregulation on T cells was further examined by linear regression. As shown in Figures 3A and 3B, the expression of IL-5 was highly correlated (*p *< 0.0001) with CD38 and CD69 expression using the mean fold change of three experiments and the four retinoids at the doses indicated in Tables 1 and 2. A similar degree of correlation between the expression of these CD markers and IL-4 levels was also observed in these studies (data not shown).

**Table 3:** ATRA, 9-cis RA and 13-cis RA significantly increase the expression of CD38 and CD69 on T cells in anti-CD3-stimulated PBMC cultures in a dose-dependentfashion.

**CD38 MCN**	**Fold vs Control ± SE (n = 3)**			
	**Dose (nM)**			

	**1**	**10**	**100**	**1000**

**Retinoid**				
**ATRA**	1.49 ± 0.07	1.60 ± 0.05	1.63 ± 0.07	1.67 ± 0.05
**9-cis-RA**	1.11 ± 0.02	1.43 ± 0.02	1.77 ± 0.08	1.93 ± 0.02
**13-cis-RA**	1.28 ± 0.06	1.50 ± 0.06	1.68 ± 0.10	1.71 ± 0.08
**4-HPR**	1.11 ± 0.02	1.15 ± 0.02	1.20 ± 0.02	1.66 ± 0.19
				
**2-way ANOVA**				
Dose	*p *= 0.0001			
Retinoid	*p *< 0.0001			
				
**Tukey Kramer**				
Dose	*p *< 0.01	1 nM vs 10 nM		
		1 nM vs 100 nM		
		1 nM vs 1000 nM		
Retinoid	*p *< 0.01	4-HPR vs ATRA		
		4-HPR vs 9-*cis *RA		
		4-HPR vs 13-*cis *RA		

**CD69 MCN**	**Fold vs Control ± SE (n = 3)**			

	**Dose (nM)**			

	**1**	**10**	**100**	**1000**

**Retinoid**				
**ATRA**	1.29 ± 11.0	1.27 ± 10.5	1.40 ± 0.14	1.46 ± 0.15
**9-*cis*-RA**	1.07 ± 3.4	1.27 ± 8.2	1.59 ± 0.16	1.64 ± 0.15
**13-*cis*-RA**	0.99 ± 4.0	1.12 ± 3.4	1.24 ± 0.08	1.33 ± 0.11
**4-HPR**	0.85 ± 6.2	0.86 ± 3.3	0.94 ± 0.04	1.08 ± 0.04
				
**2-way ANOVA**				
Dose	*p *= 0.0001			
Retinoid	*p *< 0.0001			
				
**Tukey Kramer**				
Dose	*p *< 0.01	1 nM vs 100 nM		
		1 nM vs 1000 nM		
Retinoid	*p *< 0.01	4-HPR vs ATRA		
		4-HPR vs 9-*cis*-RA		
		4-HPR vs 13-*cis*-RA		

### Differential regulation of T cell activation markers and type 1- and type-2-associated cytokines by an RAR-α agonist and a RAR-α antagonist

**Figure 4:**
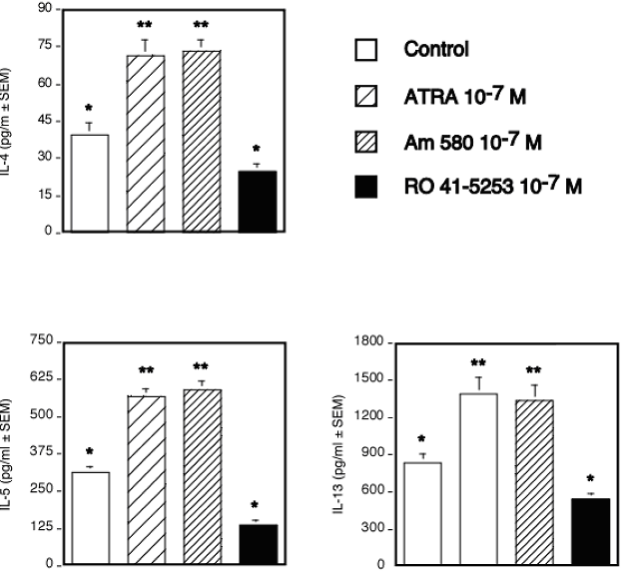
**A RAR-α agonist specifically induces Th2-associated cytokine production by anti-CD3,-activated PBMC**. IL-4, IL-5, and IL-13 proteins were quantitated by ELISA in supernatants of 48 h, anti-CD3,-activated PBMC treated with EtOH, ATRA (10^-7 ^M), the RAR-α agonist, AM580 (10^-7 ^M), or an RAR- α antagonist, RO 41-5254 (10^-7 ^M). The values shown represent the average obtained from 4 donors ± SEM. Means that express different superscripts are significantly different by at least p < 0.02.

**Figure 5:**
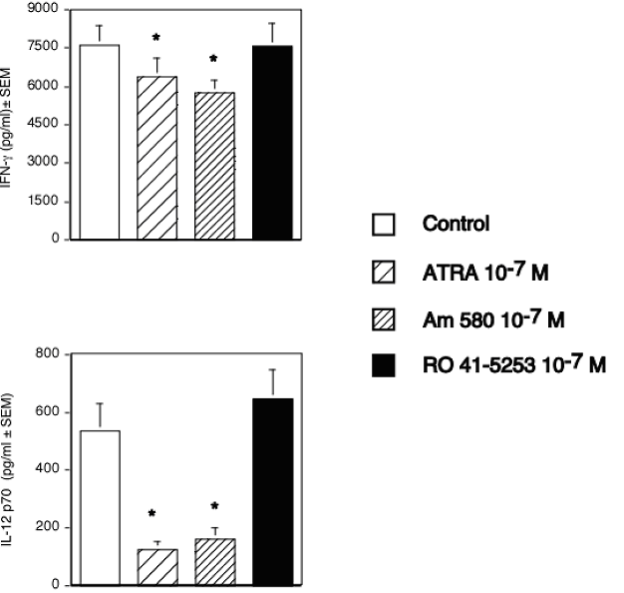
**A RAR-α agonist specifically inhibits Th1-associated cytokine production by anti-CD3,-activated PBMC**. IFN-γ (panel A) and IL-12p70 (panel B) proteins were quantitated by ELISA in supernatants of 48 h, anti-CD3,-activated PBMC treated with control EtOH, ATRA (10^-7 ^M), AM580 (10^-7 ^M), or an RAR- α antagonist, RO 41–5254 (10^-7 ^M). The values shown represent the average obtained from 4 donors ± SEM. Means that express different superscripts are significantly different by at least p < 0.03.

**Figure 6:**
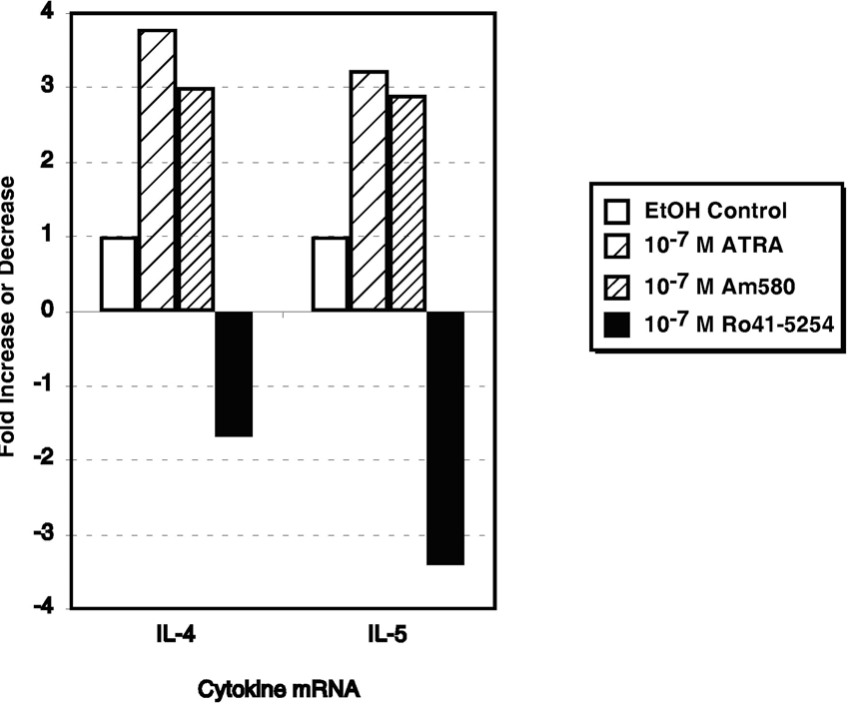
**ATRA and an RAR- -α agonist upregulate and an RAR-α antagonist downregulates the expression of IL-4 and IL-5 mRNA in anti-CD3, -activated PBMC**. Taqman^® ^semi-quantitative PCR for IL-4 and IL-5 transcripts was performed using total cellular RNA of 48 h, anti-CD3,-activated PBMC treated with EtOH, ATRA (10^-7 ^M), an RAR- α agonist, AM580 (10^-7 ^M), or an RAR- α antagonist, RO 41–5254 (10^-7 ^M). Values obtained for each cytokine message was normalized to that obtained for 18S rRNA in the same sample as described in "Materials and Methods". The normalized values were then expressed as a function of the ETOH control sample. The data are representative of all of the donors tested.

Based on the differential ability of 13-*cis *RA and 4-HPR to modify Th2-associated cytokine production and the high degree of correlation with expression of CD38, a RAR-α-induced cell surface marker, we hypothesized that RAR-α may be involved in the process of type 2 differentiation. However, the use of these retinoids could not exclude a role for other RAR and RXR receptors due to possible inter-conversion between the various retinoids. Therefore, a highly selective RARα agonist and antagonist were examined and compared with ATRA for their ability to induce type 2 cytokine production. The results in Figure 4 demonstrate that both ATRA and the RARα agonist, AM580, significantly induced IL-4, IL-5 and IL-13 synthesis above vehicle control. They had no effect on IL-10 (data not shown). In contrast, the RAR-α selective antagonist, Ro 41-5254, decreased IL-4, IL-5 and IL-13 synthesis to approximately 60% of control values; although these differences were only marginally significant (p = 0.11, 0.13 and 0.13 respectively). This is most likely due to the variability inherent in using PBMCs from different human donors. Similar to the previous ATRA results, AM580 exhibited a significant inhibitory effect on both IFN-γ and IL-12 synthesis using anti-CD3-stimulated PBMC (Figure 5). The RAR-α-antagonist exerted little to no effect on either type 1-associated cytokine production suggesting the effects of this antagonist within the culture system is specific and direct for type 2 cytokines. While not shown, both ATRA and AM580 exhibited a similar potency and efficacy in our culture systems. These retinoid-induced effects induced similar effects on type 2 cytokine mRNA expression (Figure 6).

For the sake of clarity and cohesion with regard to retinoid effects on Th1 and Th2 -associated cytokine expression and production, we have focused mainly on our PBMC data in this manuscript; however, IL-12 is likely produced by accessory cells such as monocyte/macrophages in our system. However, we have demonstrated similar enhancement of IL-4, IL-5, and IL-13 levels using AM580 and ATRA agonists and the RO 41–5254 antagonist within anti-CD3 and anti-CD28-stimulated highly purified T cell cultures (Table 4A). The statistical trends were weaker than PBMCs because of the high level of donor to donor variation in production of these cytokines. The lack of an effect on IFN-γ production probably reflects the influence of retinoids on IL-12 and other cytokines. Interestingly, IL-10 production was inhibited by ATRA and Am-580 as well as the RAR-α antagonist. Similar to the data reported for PBMCs, the effects of the retinoids on IL-4, IL-5 and IL-13 were similar to their effects on CD38 and CD69 expression (Table 4B). These results suggest that endogenous retinoids have multiple actions and may play an influential role in T cell differentiation and cytokine expression.

**Table 4:** ATRA and the RAR-α agonist, AM580, directly influence T cell activation and induce T cell differentiation towards a Th2 phenotype.

A.					
Treatment	Cytokine (pg/ml) ± SE (n = 4)				

	IL-4	IL-5	IL-13	IL-10	IFN-γ

EtOH Control	77 ± 32 ^ac^	415 ± 188 ^a^	524 ± 249^a^	491 ± 143 ^a^	4260 ± 674
ATRA	112 ± 48^ab^	775 ± 328 ^b^	671 ± 280^b^	295 ± 88 ^b^	4135 ± 379
Am 580	107 ± 34^ab^	744 ± 307 ^ab^	599 ± 300^b^	308 ± 94 ^b^	3835 ± 457
RO 41–5253	42 ± 15 ^c^	223 ± 109 ^c^	466 ± 232^ac^	295 ± 55 ^b^	3230 ± 648

(IL-10, n = 3)					
					
B.					
			
Treatment	CD Marker MCN ± SE (n = 3)				
			
	CD38	CD69			
			
EtOH Control	466 ± 60 ^a^	348 ± 41^a^			
ATRA	1073 ± 197 ^b^	521 ± 34^b^			
Am 580	1019 ± 151 ^b^	467 ± 18 ^c^			
RO 41–5253	296 ± 21 ^a^	263 ± 27 ^d^			

T cells were activated for 48 h and cytokines and surface markers were measured as described in the *Materials and Methods*. Measurements were compared with repeated measures ANOVA. Values with different superscripts are significant at p < 0.05. All retinoids were used at a final concentration of 1 × 10^-7 ^M.

These data on RAR-α agonist and antagonist cannot exclude a role for other RAR receptors in Th2 development. To this end we have also examined the comparative ability of the RAR-γ agonist SR11254 and RAR- β/γ agonist, Ro 44–5743, to stimulate Th2 cytokine syntheses in CD3,-stimulated PBMC and CD3,- and CD28-stimulated T cells. Compared to ATRA and AM580, these agonists were significantly less effective in stimulating IL-5 synthesis (data not shown).

**Figure 7:**
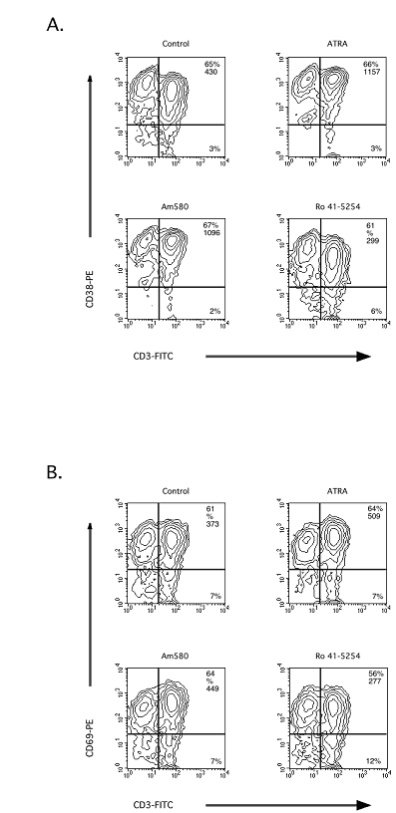
**A RAR- α agonist induces and the RAR- α antagonist, R0 41–5254, reduces the expression of CD69 and CD38 on human T cells during activation**. PBMC were activated with anti-CD3, mAb in the presence or absence of control ETOH or 10^-7 ^M of ATRA, AM580, or RO41-5254 for 48 h. After activation, the cells were harvested and the cell surface levels of CD69 and CD38 were assessed by flow cytometric analysis as described in the *Materials and Methods*. The data are representative of all of the donors tested.

Similar to the data on ATRA and 9-*cis*-RA shown in Table 3, AM580 increased the MCN and the percentage of T cells expressing CD69 (Figure 7A) and CD38 (Figure 7B) in anti-CD3, activated PBMC cultures. We typically observed a 2–3 fold increase in the MCN of CD69 and CD38 using these retinoids. Conversely, the RAR-α-selective antagonist inhibited the expression of these molecules (Figure 7A and 7B). Thus, it would appear that ATRA and the RAR-α selective retinoid, AM580, induce similar and comparable activation of human T cells in our culture system. In addition, we have also examined the comparative ability of the RAR-γ agonist SR11254 and RAR-β/γ(agonist, Ro 44–5743, to stimulate CD38 and CD69 expression in anti-CD3,- or anti-CD3/anti-CD28-stimulated PBMC and T cells. Compared to ATRA and AM580, these agonists were significantly less effective at inducing the expression of CD38 and CD69 (data not shown).

### Lack of involvement of liganded RXR in ATRA- and RAR-α-mediated cytokine production

**Figure 8:**
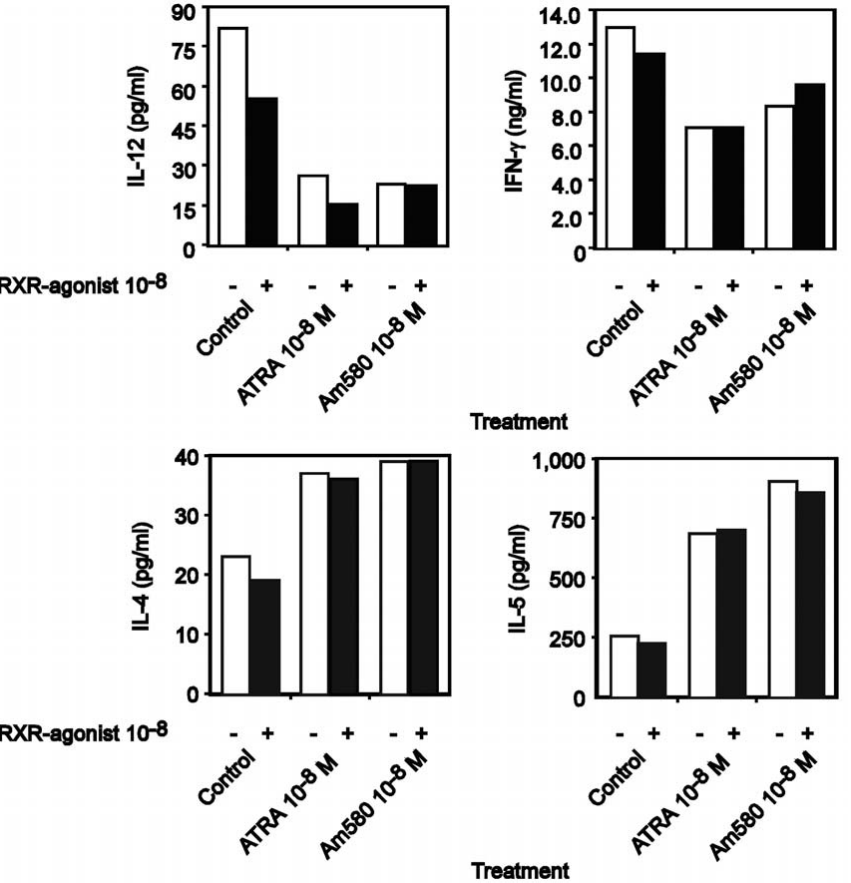
**The effects of ATRA on Th1 and Th2 cytokine production are primarily mediated through RAR-α with minimal involvement of liganded RXRs**. IL-12p70 and IFN-γ (Panel A) and IL-4 and IL-5 (Panel B) proteins were quantitated by ELISA in supernatants of 48 h, anti-CD3,-activated PBMC treated in the absence or presence of EtOH, ATRA (10^-8 ^M), or the RAR-α agonist, AM580 (10^-8 ^M) and in the presence or absence of EtOH (□) or the RXR agonist (10^-8 ^M, ■). The above data are representative of the results from two different donors examined.

Because ATRA and 9-*cis *RA were relatively equipotent in their effects on IL-4, IL-5 and IL-13 synthesis, we hypothesized that liganded RXRs may be only minimally involved in the type 2 cytokine-inducing effects of ATRA. Previous studies that examined the ability of certain retinoids to inhibit IL-12p70 synthesis and to induce thymocyte apoptosis, found 9-*cis*-RA to be at least 10 times more potent than ATRA on a molar basis (5, 23). The authors of these studies have suggested that given the enhanced effects of 9-*cis*-RA that RXRs were involved in this process [14,36]. To directly address this question, we have examined the ability of an RXR-selective retinoid, SR11345, in conjunction with ATRA or the RAR-α-selective retinoid, AM580 to modulate type 1 and 2 cytokine production in two independent human donors. Previous reports have demonstrated a molecular cooperative or obligate interactions of liganded RARs receptors with liganded RXRs by using combinations of RAR- and RXR-selective ligands [14,37,38]. The results in Figure 8 demonstrate the ability of the RXR agonist, SR11345, to inhibit IL-12p70 and IFN-γ synthesis (Panel A), but with little to no additional activity on type 2- cytokine synthesis alone or in combination with RARα agonists (Panel B). Similar results were obtained for IL-4 and IL-5 levels using purified human T cells (data not shown). These results and those in Table 2, suggest that liganded RXRs appear to exert inhibitory effects on type 1 cytokine production alone but are less efficacious than RARα agonists and do not appear to be playing a role in type 2 cytokine responses.

## Discussion

Several clinically useful retinoids including 13-*cis *RA (Isotretinoin or Accutane^®^), and 4-HPR (fenretinide), differ from ATRA (Tretinoin^®^) and 9-*cis *RA (Panretin^®^) in their biological activity and/or toxicity. These differences in activity are thought to be primarily due to their differential ability to bind RAR or RXR and their subsequent ability to induce gene transactivation and/or transrepression. 13-*cis*-RA, in conjunction with IFN-γ is an effective chemotherapeutic agent for squamous cell carcinoma of the skin and cervix and is useful in the chemoprevention of secondary head and neck tumors [13]. The RAR and RXR binding and transactivating ability of 13-*cis *RA are controversial. Several studies have suggested that 13-*cis *RA possesses a moderate affinity for RARs, especially RAR-α, but possesses little to no affinity or activating potential for RXRs [8,39-41]. However, like ATRA and 9-*cis *RA, *in vitro *conversion of 13-*cis *RA to other bioactive retinoids has been demonstrated [9]. 4-HPR, a synthetic retinoid, exerts potent chemopreventive action in animal models of carcinogenesis; however, its effectiveness in humans remains to be determined [42]. Like 13-*cis *RA, the ability of 4-HPR to bind and transactivate RARs or RXRs is a matter of controversy [43-46]. In studies examining 4-HPR interactions with these receptors, 4-HPR appears to bind to and transactivate RAR-β and RAR-γ but not RAR-α and RXRs [47]. 4-HPR does not undergo appreciable transformation to retinoids such as ATRA [42].

Several lines of evidence presented within the current report suggest that RAR-α-selective retinoids can recapitulate all of the previously observed effects of retinoids on the differentiation process of Th2 cells: (1) ATRA and 13-*cis *RA, which operate mainly via RARs, induces type 2 cytokine expression; (2) 4-HPR, a potential transactivator of RAR-β/γ(but not RAR-α or RXRs, exerts minimal effects on expression of IL-4, IL-5, and IL-13; (3) TTNPB, a selective activator of RARs, is capable of inducing cytokine expression similar to ATRA, 9-*cis *and 13-*cis *RA (H. Dawson and D.D. Taub, unpublished observation); (4) AM580 stimulates type 2 cytokine synthesis to the same degree as ATRA; (5) the RAR- α-selective antagonist, Ro 41–5254, inhibits the expression of Th2-like cytokines; and (6) the use of an RXR-selective agonist alone or in combination with ATRA or AM580, exerts no effect on type 2 cytokine expression. Together, these results suggest that in vitro human Th2 cell differentiation is predominantly regulated by retinoids through the RAR-α-dependent signaling pathway.

There are a very limited number of studies that have examined the role of specific RARs in the regulation of IL-4 production by rodent and human T cells. In one study, the pan RAR selective retinoid, TTNPB, was active, but far less effective, than either ATRA or 9-*cis *RA in inducing IL-4 synthesis from antigen-stimulated murine T cells [48]. However, the role of the RAR receptor in this regulation is unclear because the RAR-α-selective retinoid, Am-80, failed to affect IL-4 production by antigen-stimulated murine Th2 clones [23] or antigen-stimulated murine T cells [21] and *in vitro *antigen-stimulated murine T cell IL-4 synthesis was stimulated by 9-*cis*-RA and an RXR-selective retinoid, but not by ATRA or an RAR-selective retinoid [49]. The effects of receptor-selective retinoids on the production of other Th2 cytokines were not examined in these studies. The current report has directly demonstrated an RAR-α-specific effect on type 2 cytokine expression on human PBMC and T cells in the absence of accessory cells.

Our current data appear to contradict findings of two published studies where 13-*cis*, ATRA, and 9-*cis*-RA were all equipotent in inhibiting IFN-γ production from rodent Th1 cells [25]. In addition, *in vitro *antigen-stimulated murine T cell IL-4 synthesis was stimulated by 9-*cis*-RA and an RXR-selective retinoid, but not by ATRA or an RAR-selective retinoid [49]. Our data regarding RAR-α also differ with those of another report where an experimental RAR-α-selective retinoid failed to affect the production of IFN-γ by Th1 clones and IL-4 by mouse Th2 T cell clones [23] or antigen-stimulated naïve T cells [21]. In a more recent study, ATRA, 9-*cis*-RA, and an RAR agonist were found to be potent inducers of cellular proliferation and IL-2 production by enriched human T cells [50]. These effects were found to occur predominantly with RAR agonists and only marginal effects being observed through the use of RXR agonists. More interestingly, while we have also observed increased activation and proliferation by human T cells upon culture with RAR agonists, no significant changes in the production of IL-2 (another type 1 cytokine) were observed post RAR agonist treatment in our hands (data not shown). In addition to authentic species and differences in biological activities of synthetic retinoid putatively acting through the same receptor, simple differences between cell purification procedures, culture conditions, activation regiment, age of donors, memory/naïve and CD4/CD8 ratios, nutritional status, species and the donor population being examined may account for differences between studies. However, we have found similar results with ATRA, 9-*cis-*RA and Am580 when using pig PBMCs (H. Dawson, unpublished).

One potential limitation of our study is the relatively weak antagonistic activity demonstrated by the RAR-$\tilde{\alpha }$ antagonist, Ro 41–5254. This antagonist binds to RAR-α with four and two times less affinity than ATRA and AM580, respectively [51]. Previous studies have effectively utilized this compound only at a molar ratio of 1000:1 to 100:1 of antagonist to agonist [35,51]. Using the inhibition of induction of CD38 antigen expression by ATRA as a bioassay for RAR-α-mediated activation, this compound exhibited a 10%, 40%, and 80% inhibitory activity at a molar ratio of 10:1, 100:1, and 1,000:1, respectively (data not shown). Using a 100:1 molar ratio of antagonist to agonist (AM580 or ATRA), we have also observed a 60–70% reduction in the synthesis of Th2-associated cytokines by CD3-stimulated PBMC (data not shown). There is recent evidence that Ro 41–5254 is a peroxisome proliferator-activated receptor-gamma (PPAR-γ) agonist [52]. PPAR-γ agonists can inhibit Th1 and Th2 responses. Although Ro 41–5254 exhibited little activity in this regard in our system, we cannot rule out interactions between the retinoid and PPAR pathways. Another limitation is that we cannot rule out the possibility that there was sufficient endogenous RXR-active retinoids in this system to permit RAR- and liganded RXR-mediated nuclear transactivation. However, as normal serum (similar to the serum utilized in our culture system) contains negligible concentrations of 9-*cis *RA but appreciable quantities of other retinoids such as ATRA (10 to 100 nM) and 13-*cis *RA (2.5 to 25 nM), we feel that this is unlikely.

RAR-α has at least two isoforms, RAR-α-1 and RAR-α-2, which arise as a consequence of differential splicing. Analysis of transgenic mice, which under- or overexpress RAR-α-1, has revealed limited effects on the development, phenotype and/or function of the immune system [53]. Adult mice expressing low amounts of RAR-α develop spontaneous *T *and *B *cell lymphomas and had very low amounts of the RA-inducible gene, CD38, in thymus and bone marrow [35,54]. *In vivo *experiments have suggested that RAR-α agonists may exhibit inhibitory effects on T-cell mediated immunity. Systemic administration of RAR-α-selective retinoids have been shown to inhibit DTH [21,29], the progression of experimental arthritis [23], prolong skin allograft survival [30], and inhibit Ab production in mice [29].

Our proposed model of the role of RAR-α in Th2 development has parallels in other cell types. Recent studies suggest that the differential engagement of individual RARs or RXRs may regulate the process of monocyte differentiation into macrophages or dendritic cells [55] and the induction or prevention of apoptosis of B cells and T cells [14,56], as well as normal neutrophil differentiation [57]. Several recent studies have focused on the effects of receptor-selective retinoids on accessory cells in the regulation of Th1 or Th2-like cytokine production by T cells. 9-*cis*-RA and the RXR-selective retinoid, LG69, have been shown to inhibit LPS-stimulated IL-12 production from murine macrophages more effectively than the pan RAR-selective retinoid, TTNPB [39]. Similarly, 9-*cis*-RA was more effective than TTNPB in downregulating IL-12 protein synthesis in LPS- or KLH-stimulated mouse macrophages [48]. More recent human studies have demonstrated a direct effect of ATRA and RXR rexinoids on IL-2 receptor expression by human cutaneous T-cell lymphomas through RAR and RXR receptors (45,46). Overall, the bulk of evidence from all of these studies point to a predominant role for liganded RXRs in these processes.

## Conclusion

Our data suggest a lesser role for liganded RXRs in the inhibition of IFN-γ and IL-12 production in a mixed population of cells during T cell activation. A dose-response titration of the RXR-selective retinoid showed that it was approximately 100 fold less effective than ATRA and 9-*cis*-RA and 10 times less effective than the pan RAR agonist TTNPB in inhibiting IFN-γ and IL-12 expression (H. Dawson and D.D. Taub, unpublished observations). These findings are in direct contrast with a recently published murine T cell study demonstrating that stimulation of the RXR pathway enhances Th2 development by murine T cells (47). While it would appear, based on the published retinoid literature, that human and murine T cells differentially respond to RAR and RXR agonists, we believe that the inactivity of the RXR ligand in regulating the production of Th2 cytokines by human T cells suggests only the involvement of the RAR/RXR heterodimer and not the RXR/RXR homodimer in Th2 cytokine induction.

## Methods

### Reagents

ATRA, 9-*cis *RA, and 13-*cis *RA were purchased from Sigma (St Louis, MO), the RAR-α agonist, AM580 (Ro-40–6055) [58], 4-HPR, and the pan RAR agonist TTNPB were purchased from Biomol (Plymouth Meeting, PA). The RAR-α antagonist, Ro 41–5253 [51], and RAR-γ agonist, Ro 44–5743, were generously provided by Michael Klaus Ph.D. (Hoffman LaRoche, Basel) and the RXR agonist, SR11345 [59] and RAR-γ agonist, SR11254 [60], were generously provided by Marcia Dawson Ph.D (Medicinal Chemistry Department, Molecular Medicine Research Institute, Mountain View, CA). The retinoids were pre-diluted 1000X in ethanol and added to the media at 1 uL/mL. To avoid photo and chemical isomerization, retinoid solutions were overlayered with argon gas and stored at -80°C in the dark until use.

### Cell Preparation

Whole blood was acquired from healthy human volunteers between the ages of 21–55 years. PBMC were isolated by Ficoll Paque (Amersham Pharmacia Biotech, Piscataway, NJ) density gradient centrifugation followed by treatment with ammonium chloride (ACK) lysis solution (Biofluids, Gaithersburg, MD) to eliminate the remaining erythrocytes. The isolated cells were subsequently washed 2 times in PBS and resuspended in RPMI 1640 (Biofluids) supplemented with 10% heat-inactivated FBS (Sigma), 2% heat-inactivated pooled human AB serum (Sigma), 50:M mercaptoethanol (Gibco BRL Gaithersburg, MD), 1 mM sodium pyruvate (Biofluids), 2 mM glutamine, 1 × non-essential amino acid solution (Biofluids), 1 mg/ml gentamicin (Biowhittaker, Walkersville, MD), 100 U/ml penicillin (Biofluids), 100:g/ml streptomycin (Biofluids), and 20 mM HEPES buffer (Biofluids). T cells were isolated by negative selection using enrichment columns according to manufacturer's instructions (R & D Systems). These cells were typically > 95% pure as assessed by flow cytometric analysis. The contaminating cell population was largely CD8^+ ^and most likely were NK cells based on their size and granularity.

### Cell Culture and Harvest

PBMC (2.5 × 10 ^6 ^cells/ml) were activated with 200 ng/ml of immobilized anti-CD3ε (OKT-3, Ortho, Raritan, NJ) in the presence or absence of 0.001 to 1:M of various retinoids or EtOH vehicle control for 48 h. IL-2 (Teceleukin, Hoffman LaRoche, Nutley, NJ) at 10 U/ml or 1:g/ml of neutralizing anti-cytokine mAb were added to the cultures where indicated. Alternately, T cells (1.0 × 10 ^6 ^cells/ml) were activated with 200 ng/ml of immobilized anti-CD3, and 1:g of soluble anti-CD28 (clone 28.2, Pharmingen) or with IL-2 at 10 U/ml where indicated. PBMC or T cells were harvested at various time intervals after incubation at 37° and 5% CO_2_. Non-adherent cells were decanted from the flasks and centrifuged to obtain supernatants. The flasks were then treated with Enzyme-Free cell dissociation solution (Specialty Media, Phillipsburg, NJ) and were gently scraped to remove and harvest cells. Viable cells from the decanted cells and cell removal mixture were isolated by Ficoll Paque density gradient centrifugation as above.

### Cytokine ELISA

ELISAs (Biosource) were utilized to examine the following human-specific cytokines: IFN-γ, IL-2, IL-4, IL-5, IL-10, IL-12 p70 and IL-13. All of the ELISAs were performed according to the manufacturer's instructions. The results are expressed as pg/ml or ng/ml and all assays were run in duplicate with at least three separate experiments being examined.

### Real Time PCR

Cytoplasmic RNA was extracted and purified using a commercially available kit (RNAeasy, Qiagen, Valencia, CA). Purified RNA was electrophoresed on a 1% agarose gel to assess the integrity of the purified RNA. One μg of RNA was reverse transcribed into cDNA using a commercial available kit (Applied Biosystems, Foster City, CA). One hundred pg RNA equivalent of this cDNA was used for PCR amplification. PCR reactions were performed in special optical tubes in a 96 well microtiter plate format on an ABI PRISM 7700 Sequence Detector System (PE Applied Biosystems) using pre-developed FAM- and TAMRA-labeled internal oligonucleotide probes and primers for IL-4 and IL-5 (PE Applied Biosystems). Each reagent also contains VIC- and TAMRA-labeled internal oligonucleotide probes and primers specific for the 18S RNA ribosomal subunit. Amplification conditions were as follows 25°C for two min; 95°C for 10 min; 40 cycles of 95°C 15 s and 60°C for 1 min. Fluorescence signals measured during amplification were processed post-amplification and were regarded as positive if the fluorescence intensity was ten fold greater than the standard deviation of the baseline fluorescence. This level is defined as the threshold cycle (Ct). The Ct value for18S ribosomal subunit was subtracted from the Ct value for each cytokine message to normalize for RNA content. This value is defined as ΔCT. To evaluate the effects of retinoids, ΔCT_treatment _was subtracted from ΔCt_control_. This value is defined as ΔΔCT. The relative folds increase or decrease was then calculated as 2^-ΔΔCT^.

### Flow Cytometric Analysis

Anti-human CD3-FITC (Pharmingen, San Diego, CA), anti-human CD25-PE, CD38-PE, and, CD69-PE (Becton Dickinson) were used to assay cell purity and activation status. Cells (0.25 × 10^6^) were suspended in 50:L of staining buffer (1% FCS, 1% goat serum, 2.5:g of mouse IgG/50:L) in round-bottom 96-well plates and incubated at 4°C for 15 min. 5:L of the appropriate dilution of each antibody was then added to the appropriate wells and then incubated for 30–40 min at 4°C. After incubation, the plates were centrifuged and the cells were washed twice with 100:L of PBS/FBS buffer. After the last wash, cells were fixed in 100:L of 1% paraformaldehyde solution in PBS (Electron Microscopy Services, Fort Washington, PA, in PBS). Samples were subsequently analyzed on a FACScan flow cytometer (Becton Dickinson) and the data were processed using the Cellquest program (Becton Dickinson). A minimum of 10,000-gated events were analyzed for each sample. Data are expressed as the % of T cells expressing the marker of interest or the mean channel number (MCN) of the marker's fluorescent intensity.

### Statistical Analysis

To compare the effects of retinoids at various concentrations a two factor ANOVA was performed. Data were first expressed as percentage of vehicle control culture and then analyzed for equality of variance using Fisher's F test. If the variance was heterogeneous, the appropriate transformation of the data was performed. A two factor ANOVA was used to analyze the effect of retinoid, dose or any interaction between retinoid and dose. If no significant interaction between retinoid and dose was present in the two-way ANOVA, a Tukey-Kramer post-hoc analysis was performed to determine statistically significant differences between each factor level of retinoid and dose. To examine correlation between the level of T cell activation and cytokine production, simple regression was performed using activation markers as the independent variable and cytokine levels as the dependent variable. To evaluate the comparative effects of ATRA, an RAR agonist and RAR-α antagonist, a repeated measures ANOVA was used after data were examined for equality of variance as above. A *P *< 0.05 was considered statistically significant for all analysis. All analysis was performed using Statview 5.0 for Macintosh (Abacus Concepts, Berkeley, CA).

## List of Abbreviations

ATRA: all-*trans *retinoic acid; 9-*cis *RA: 9-*cis *retinoic acid; DTH: delayed type hypersensitivity; pTh: precursor T helper; RARs: retinoic acid receptors; RXRs: retinoid × receptors; VA: Vitamin A; 4-HPR: 4-hydroxyphenylretinamide; TTNPB: 4- [2-(5,6,7,8-tetrahydro-5, 5,8,8-tetramethyl-2-naphthalenyl)-1-propenyl]benzoic acid; Vit D_3_: 1, 25 (OH)_2 _vitamin D_3._

## Authors' contributions

HD, GC, RP, MK and DDT did the experiments. HD and DDT prepared the figures and co-wrote the paper. DDT supervised the work and edited the manuscript.
